# SGLT2 inhibitor administration to two patients with diabetes mellitus with ascites due to cirrhosis

**DOI:** 10.1530/EDM-25-0015

**Published:** 2025-07-24

**Authors:** Koji Nagayama, Risako Harada, Hiroshi Ajima, Sakurako Orikasa, Misaki Aoshima, Yutaka Oki

**Affiliations:** ^1^ Department of Endocrinology, Diabetes, and Metabolism, Hamamatsu Medical Center, Hamamatsu, Shizuoka, Japan; ^2^ Center of Diabetes and Endocrinology, Hamamatsu-Kita Hospital, Hamamatsu, Shizuoka, Japan

**Keywords:** cirrhosis, ascites, diabetes mellitus, sodium-glucose cotransporter 2 inhibitor

## Abstract

**Summary:**

We used the sodium-glucose cotransporter 2 inhibitor, luseogliflozin in two patients with diabetes mellitus with Child–Pugh classification B cirrhosis and cirrhotic ascites. In each case, luseogliflozin was safely used for over three years and was also considered effective in reducing ascites. In one of the patients in particular, when luseogliflozin was discontinued and switched to insulin treatment before colorectal cancer surgery, ascites accumulation was observed within two weeks, which subsequently decreased rapidly when luseogliflozin was restarted. In this case, the effect of luseogliflozin on ascites was evident by the clear increase and decrease in ascites over a short period of time, as evaluated using body weight, abdominal circumference and CT scan, without changing her other diuretic medication. Although sodium-glucose cotransporter 2 inhibitors need to be used with caution, they might be an option for the treatment of diabetes in patients with cirrhosis.

**Learning points:**

- Luseogliflozin, a sodium-glucose cotransporter 2 inhibitor, is effective for glycemic control and safe in patients with cirrhosis.
- Luseogliflozin administration reduced ascites in patients with diabetes mellitus.
- Caution is warranted, as discontinuation of sodium-glucose cotransporter 2 inhibitors might lead to an increase in ascites.

## Background

Conventionally, insulin has been the main treatment for diabetes in patients with liver cirrhosis. However, patients with diabetes mellitus with cirrhosis often require large amounts of insulin. High-doses of insulin are known to have proliferative effects on human hepatocytes, and it has been suggested that hepatic carcinogenesis in chronic liver disease might possibly be induced by insulin treatment (1). Moreover, many anti-diabetic drugs other than insulin are contraindicated and require careful dose-adjustment in patients with liver cirrhosis, making the selection of a suitable drug for diabetes treatment in these patients challenging. Although metformin has been reported to be effective in treating diabetes in patients with liver cirrhosis (2), in Japan, it is currently contraindicated in cases with severe hepatic dysfunction due to concerns about the risk of lactic acidosis. In addition, it is often difficult to control ascites in liver cirrhosis, and a combination of multiple diuretics is required, potentially leading to electrolyte imbalance and hyperuricemia. Sodium-glucose cotransporter 2 inhibitors (SGLT2is), which are used for the treatment of diabetes, are also reported to have diuretic properties (3). We report here the beneficial effect of administration of luseogliflozin, an SGLT2i, in two patients with diabetes mellitus and liver cirrhosis who had ascites despite the use of diuretics. Luseogliflozin was selected from among several SGLT2is because it has been reported that administration of luseogliflozin to Japanese patients with Child–Pugh class B cirrhosis is not associated with a significant change in its concentration in blood (4).

## Case presentation, investigation, treatment, outcome and follow-up

### Case 1

**Figure 1:**
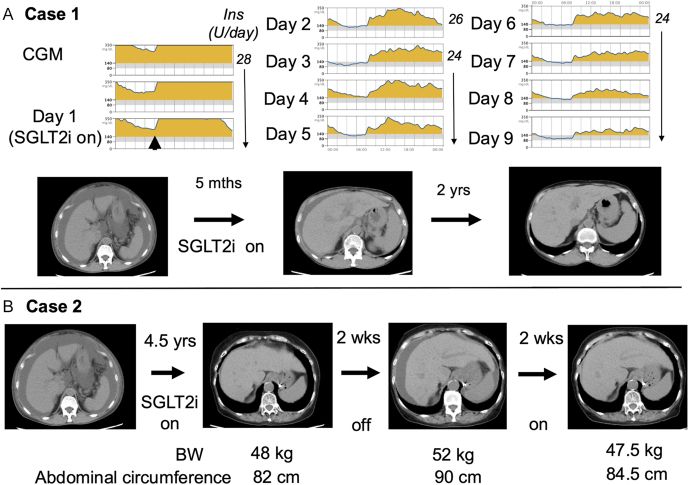
Time course of the results of continuous blood glucose monitoring and CT scan imaging in the two cases.

A 56-year-old woman, 153 cm tall, weighing 57.1 kg, with a BMI of 24.3 kg/m^2^ (on first admission), was admitted to our hospital due to ascites and cirrhosis secondary to hepatitis B infection. She had no history of hospital visits until then. The ascites was treated with the combined use of multiple diuretics (furosemide 20 mg/day, spironolactone 25 mg/day and tolvaptan 7.5 mg/day) and albumin infusions (25% 50 mL/day, 3 times). She was also diabetic, and her fasting blood glucose remained above 11 mmol/L despite receiving 28 units of insulin/day as part of intensive insulin therapy. Her fasting blood glucose levels rapidly decreased from the day after commencing administration of luseogliflozin 5 mg/day, improving to approximately 7 mmol/L without altering her other treatment methods. Diuretics and albumin infusion resulted in a 4 kg weight loss over 10 days, and an additional 3 kg weight loss was seen within a week after starting the luseogliflozin administration, with most of this overall weight loss assumed to be due to ascites reduction. Upon discharge, her insulin dosage was reduced to 24 units/day. A continuous glucose monitor (CGM) was used during this period to ensure that no episodes of hypoglycemia occurred (Fig. 1). Following discharge, she maintained good glycemic control with a decrease in ascites and was able to reduce diuretic usage. For hepatitis B, entecavir (0.5 mg/day, oral administration) was started 3 months after discharge and has been continued since then. The use of luseogliflozin 5 mg/day was continued safely for over 3 years. Her Child–Pugh grade improved from B to A (Table 1). After the initial weight change associated with decreased ascites, she did not show significant weight loss or muscle weakness. She was able to continue SGLT2i treatment without any adverse effects.

**Table 1:** Characteristics of the two patients with diabetes mellitus with cirrhosis and treated with the SGLT2 inhibitor.

	Case 1			Case 2		
	Before	3 months	3 years	Before	3 months	3 years
Child–Pugh grade (score)						
A			5			6
B	9	8		9	7	
Diuretics (mg)						
Furosemide	20	20	10	60	20	
Spironolactone	50	50	25		50	25
Tolvaptan	7.5	7.5	7.5	7.5	7.5	7.5
Thiazide				1.5		
Diabetic treatment						
DPP4 inhibitor	✓	✓	✓	✓	✓	✓
Insulin	28U	14U	12U			16U
SGLT2 inhibitor		✓	✓		✓	✓
Sulfonylurea				✓		
HbA1c (%)	8.9	6.2	6.0	7.0	9.0	7.9
Weight (kg)	57	41	39	50	41	47
ALT (U/L)	36	69	32	12	31	36
Na (mEq/L)	134.3	134.8	141.8	140.1	136.8	140.1
K (mEq/L)	3.7	3.8	4.8	3.3	4.4	4.2
Total bilirubin (mg/dL)	2.27	2.06	0.38	1.43	0.94	0.58
Albumin (g/dL)	2.1	2.7	4.5	2.7	3.2	3.0
PT-INR	1.46	1.11	1.01	1.39	1.14	1.10
Platelets (×10^4^/μL)	9.7	12.1	16.4	10.8	13.1	11.6
eGFR (mL/min/1.73 m^2^)	85	78	66	53	60	56

### Case 2

A 73-year-old woman, 151 cm tall, weighing 50 kg, with a BMI of 22 kg/m^2^ (on initial admission), was diagnosed with diabetes at the age of 65 years and was treated with glimepiride at 1 mg/day and sitagliptin at 50 mg/day. She also had chronic rheumatoid arthritis since the age of 70 years and was under treatment with salazosulfapyridine, methotrexate and tacrolimus, resulting in stabilization of her condition. She was also found to have liver dysfunction at the age of 70 years, at which time biopsy revealed a fatty liver with no fibrosis or inflammation, although blood test results suggested autoimmune hepatitis or primary biliary cholangitis. At the age of 73 years, she was admitted to our hospital due to liver cirrhosis and ascites. During her hospitalization, she received treatment with multiple diuretics and albumin infusions for the ascites. At that time, she was on glimepiride and sitagliptin for diabetes. However, since glimepiride is metabolized by the liver, its levels might increase when administered to patients with severe hepatic dysfunction, with the potential risk of hypoglycemia. In Japan, glimepiride is contraindicated in patients with serious hepatic dysfunction according to the package insert. Therefore, glimepiride was discontinued and she was instead given luseogliflozin 5 mg/day, since it is not contraindicated in patients with severe hepatic dysfunction according to the package insert, and no significant changes in its blood concentration have been reported in patients with hepatic dysfunction (4). She continued taking a dipeptidyl peptidase IV (DPP4) inhibitor (DPP4i), linagliptin 5 mg/day, thereafter. In addition, methotrexate for rheumatism was stopped due to ascites accumulation, and the treatment was instead switched to prednisolone 5 mg/day. After discharge from the hospital, because of elevated blood glucose levels likely due to continued steroid therapy, a long-acting insulin (insulin degludec, 16 units per day) was also required. Minimal ascites was observed on CT at 6 months, and it had completely resolved on CT performed 3 years later. Along with the resolution of ascites, her Child–Pugh grade improved from B to A within 3 years (Table 1). At the age of 77 years, she was incidentally found to have colorectal cancer. In preparation for surgery, both the SGLT2i and DPP4i were discontinued, and she was switched to insulin therapy alone (54 units per day). However, after discharge, she noticed rapid weight gain, edema and abdominal distention, and CT scan showed ascitic effusion (Fig. 1). Hence, she was readmitted and luseogliflozin 5 mg/day and linagliptin 5 mg/day were restarted, while the insulin dosage was reduced to 44 units per day. This treatment approach led to a decrease in ascites (Fig. 1), allowing surgery for the colorectal cancer to be performed successfully. The SGLT2i was discontinued for 3 days before and after the surgery, and the patient was able to undergo the surgery without re-accumulation of ascites.

## Discussion

Although there are only a few reports of the use of SGLT2is for the treatment of diabetes in cirrhotic patients with ascites (5, 6), these agents have been reported to be effective for the treatment of both diabetes and ascites in such cases (7). In our cases, multiple treatments for ascites were initiated simultaneously; however, SGLT2is were introduced after other diuretics, enabling dose reductions of those agents and suggesting an independent therapeutic effect of SGLT2is on ascites. In particular, the beneficial effect of the SGLT2i on ascites in case 2 was supported by the rapid worsening of ascites following preoperative discontinuation of the SGLT2i, despite no changes in other treatment modalities.

When using SGLT2is in patients with diabetes mellitus and cirrhosis, it is necessary to pay attention to the risk of hypoglycemia due to the reduced amount of glycogen accumulation in the liver. As demonstrated using a CGM in case 1, the SGLT2i was found to be effective from the first day of its administration. Considering the increased risk of hypoglycemia in cirrhotic patients, those on insulin therapy require a CGM and appropriate insulin dose reduction with the start of SGLT2i treatment. In the present cases, SGLT2i and insulin were used concomitantly, but with careful self-monitoring of blood glucose, resulting in no episodes of severe hypoglycemia. In case 1, the insulin dose was not reduced, as the SGLT2i was introduced during a state of hyperglycemia. In general, when glycemic control is adequate, a reduction in insulin dosage by approximately 20% has been suggested (8). However, in patients with cirrhosis, insulin titration should be approached with greater caution, accompanied by close monitoring of blood glucose levels.

In addition, since SGLT2is can cause frailty, it is necessary to carefully observe the patients for maintenance of body weight and changes in body composition, especially in those with decompensated cirrhosis. Although body composition changes were not routinely assessed in the present cases, their physical fitness and activity levels were assessed as a minimal but essential measure, to determine the appropriateness of the observed weight changes.

Especially in patients with ascites, since changes in body weight might also be due to an increase or decrease in ascites, it is necessary to evaluate ascites by measuring abdominal circumference and body weight concurrently.

The use of diuretics in the treatment of liver cirrhosis often presents problems in terms of electrolyte abnormalities, which might affect the prognosis. In both the present cases, ascites decreased after the administration of the SGLT2i, allowing the dose of furosemide to be reduced, with a resultant improvement in hypokalemia. SGLT2is have been reported to exert minimal effects on serum potassium levels, which might confer an additional clinical advantage of these drugs (9).

In addition, in patients with cirrhosis, careful attention should be paid to the risk of hepatic encephalopathy secondary to elevated ammonia levels. In both our cases, serum ammonia levels were regularly monitored and remained within the normal range, including after the initiation of SGLT2i therapy.

Although SGLT2i administration in patients with decompensated cirrhosis requires careful attention, it provides good glycemic control, facilitating the avoidance of high insulin doses, and might have a positive effect on ascites. A previous report using a propensity score-matched intention-to-treat analysis demonstrated that SGLT2is might improve survival compared with DPP4is in cirrhotic patients receiving metformin (10). The authors noted that over 40% of their cohort had NAFLD-related cirrhosis and speculated that reduced cardiovascular and oncologic mortality might contribute to the observed benefit. Furthermore, a review of several case reports suggested that SGLT2is might also be effective in patients with cirrhosis and ascites (7). SGLT2is have been reported to improve ascites control through natriuresis and osmotic diuresis without major electrolyte disturbances, potentially reducing complications such as fluid overload and hepatorenal syndrome (7). These multifactorial effects may help explain the improved outcomes in cirrhotic patients using an SGLT2i. These reports and our experience with the two cases presented here suggest that SGLT2is are likely to be beneficial in patients with diabetes mellitus with ascites and cirrhosis. However, like other diuretics, discontinuation of SGLT2is at some point, whether due to advanced disease stage or decreased appetite due to aging, might lead to an increase in ascites. Considering that SGLT2is cause a significant change in ascites in a short period of time, as seen in case 2, determining the timing of discontinuation of SGLT2is in such cases can be challenging.

## Conclusion

While the administration of SGLT2is requires careful monitoring in cirrhotic patients, they might be a therapeutic option in patients with diabetes mellitus who also have ascites.

## Declaration of interest

KN received lecture fees from Novo Nordisk Pharma Ltd., Sanofi, Sumitomo Pharma Co., Ltd. and Mitsubishi Tanabe Pharma Corporation. RH, HA, SO, MA and YO declare that they have no conflicts of interest. This report was supported by Taisho Pharmaceutical Co. Ltd. The authors retained full control of the manuscript content.

## Funding

This research did not receive any specific grant from any funding agency in the public, commercial or not-for-profit sector.

## Patient consent

Written informed consent was obtained from the patients’ families for the publication of clinical details and clinical images.

## Author contribution statement

KN wrote the manuscript and prepared the figures and tables. RH, HA, SO and MA provided clinical care for the patients. YO critically revised the article for important intellectual content.
